# 

**Published:** 1903-10-31

**Authors:** 


					THE HOSPITAL. " The Standard Of highest purity."?Lancet. October 31st, 1903.
CADBURY'S fc* CADBURY'S
COCOA JL <??!> JP> COCOA
"A PERFECT FOOD." NO DRUGS OR ALKALIB8 UUD.
No. 892. Vol. XXXY. [gXe*8] Saturd^
Oct. 31st, 1903.
[SmbBorLPelpn7rrms'] PRICE THREEPENCE.

				

